# Potential bias in the analysis of prenatal treatment for congenital toxoplasmosis

**DOI:** 10.1371/journal.pntd.0012952

**Published:** 2025-07-07

**Authors:** Marta Gutiérrez-Valencia, Luis Carlos Saiz, Juan Erviti

**Affiliations:** 1 Innovation and Organization Unit, Navarre Health Service. Pamplona, Spain; 2 Navarre Institute for Health Research (IdiSNA). Pamplona, Navarre, Spain.; Centro de Pesquisa Gonçalo Moniz-FIOCRUZ/BA, BRAZIL

We have read the article by Guarch-Ibáñez et al. [1] with great interest and wish to contribute to the discussion on the role of prenatal treatment.

It should be noted that the authors have not taken into account certain factors, such as the existence or absence of prenatal screening, which may introduce bias into the results by overestimating the effect of treatment. According to their own results, prenatal screening was performed in 53.3% hospitals [2]. Nevertheless, this strategy identified 92.8% of congenital toxoplasmosis (CT) cases. This suggests that, in the absence of screening, far fewer cases are identified, as asymptomatic cases are not detected. The diagnosis of CT cases that have not undergone prenatal screening is made on the basis of presenting symptoms. In turn, in the absence of screening, it is not feasible for these children to have received prenatal treatment. This implies that the ‘no treatment’ group, including all cases identified without screening, is characterised by a higher prevalence of symptomatic cases, irrespective of the potential effect of treatment. The same is true for any cases of CT that were not diagnosed during pregnancy, with or without screening: such cases are probably identified for being symptomatic, and were unable to receive prenatal treatment. If the pregnant women had received a treatment with no effect, such as a placebo, differences in the clinical outcomes of the children would also have been found.

Upon repeating the analysis showed in Table 3 excluding the seven cases of CT diagnosed postpartum, assuming they were all symptomatic, the statistically significant association between prenatal treatment and clinical symptoms in children is no longer found: 33.3% without treatment (4/12) vs 25.0% with treatment (7/28); OR 1.50 (95% CI 0.344 to 6.549). The same conclusion can be reached if we exclude the four CT cases where the authors confirm that the mothers had not undergone serological screening and were identified due to symptoms. While these estimates may be inaccurate and analyses unadjusted, they serve to exemplify how the potential biases raised can strongly modify the estimated effect of prenatal treatment.

One of the most important systematic reviews on this topic performed a meta-analysis of individual patients’ data from European cohorts with 550 children born alive with congenital toxoplasmosis identified only by prenatal or neonatal screening, thus avoiding the potential bias discussed above [3]. They found no evidence that prenatal treatment significantly reduced the risk of clinical manifestations (adjusted OR for treated vs not treated 1.11, 95% CI 0.61 to 2.02). Despite being published a few years ago, SYROCOT remains a key reference document on congenital toxoplasmosis and the potential impact of prenatal treatment on clinical manifestations in children. No study of a comparable sample size and methodological quality has been conducted since its publication to contradict its conclusions.

A significant constraint on research into the treatment of congenital toxoplasmosis is the low prevalence of the disease, which presents considerable challenges in assembling cohorts with sufficient cases to draw meaningful conclusions. An interpretation of results derived from small and heterogeneous cohorts as the one presented by Guarch-Ibáñez et al. is highly limited, if possible at all [1].

Other common biases in observational studies on the efficacy of antenatal treatment include bias related to gestational age at diagnosis and at treatment initiation, as well as the interval between diagnosis and treatment [4]. The REIV-TOXO study incorporated gestational age at diagnosis into their multivariate analysis (although the trimester of maternal infection could not be determined in approximately 10% of cases), but did not consider the interval between maternal infection and prenatal treatment. The significance of this issue lies in the fact that the risk of transmission and the clinical manifestations associated with it are strongly linked to gestational age at infection. Additionally, the treatment practices employed also vary considerably depending on the gestational age as the majority of untreated women diagnosed are infected in late pregnancy. On the other hand, this study does not provide information on the efficacy of prenatal treatment in preventing maternal-fetal transmission, as it is based on information on infected children, and does not include information on infected pregnant women with uninfected children.

In our opinion, the article by Guarch-Ibáñez et al. is a valuable contribution, providing highly useful information on the epidemiology of congenital toxoplasmosis in Spain and its clinical management [1]. However, given the aforementioned limitations and the small size of this heterogeneous sample, this study is not able to support the authors’ conclusions on the efficacy of prenatal treatment.
